# Bibliography

**Published:** 1892-02

**Authors:** 


### Bibliography.

Lippincott's Monthly Magazine for January is a valuable number. "The Passing of Major Kilgore" (told by the City Editor), by Young E. Allison, is a story by one evidently familiar with newspaper life, and it, therefore, gives very vivid impressions and experiences therein.

Two fine portraits are given, one of Agnes Huntington and the other of Sydney Woollett, the reader, or "Interpreter," as Julian Hawthorne prefers to call him, in his very interesting account of Woollett and his work, which accompanies it.

TRANSACTIONS OF THE AMERICAN DENTAL ASSOCIATION. Thirty-first Annual Session, held at Saratoga Springs, New York, commencing August 4, 1891.

This exceedingly neat volume of two hundred and seventy-one pages contains the proceedings of this annual gathering. It is not too much to say that but few, if any, of the previous meetings of this association can compare with it in the presentation of papers of solid scientific value. The contents are well worth preservation in permanent form for careful reading and reference.

THE INTERNATIONAL JOURNAL OF SURGERY, New York City, opens the new year with its fifth volume.

There is no change in the editorial department, and the journal will, therefore, be conducted with the same marked ability which has heretofore governed its pages.

THE SANITARIAN, Brooklyn, N. Y.

This journal enters its twentieth year in 1892. It is "devoted to the promotion of the art and science of sanitation, mentally and physically, in all their relations, by the investigation, presentation, and discussion of all subjects in this large domain as related to personal and household hygiene."

This extensive ground is ably covered in this journal. The subjects of which it treats are of peculiar importance to the dentist, who is doomed to a sedentary life and requires the best sanitary conditions. He, above all others, should make this subject one of careful study.

